# Conservative treatment of breast ductal carcinoma in situ: results of an Italian multi-institutional retrospective study

**DOI:** 10.1186/1748-717X-7-177

**Published:** 2012-10-25

**Authors:** Cristiana Vidali, Orazio Caffo, Cynthia Aristei, Filippo Bertoni, Alberto Bonetta, Marina Guenzi, Cinzia Iotti, Maria Cristina Leonardi, Salvatore Mussari, Stefano Neri, Nicoletta Pietta

**Affiliations:** 1 S.C. Radioterapia Oncologica, Azienda Ospedaliero-Universitaria “Ospedali Riuniti”, Via Pietà 19, 34139, Trieste, Italy; 2 U.O. Oncologia Medica Ospedale S.Chiara, Largo Medaglie d’Oro 9, 38100, Trento, Italy; 3 S.C. Radioterapia Oncologica, Azienda Ospedaliera-Università degli Studi di Perugia, Località S.Andrea delle Fratte, 06156, Perugia, Italy; 4 U.O. Radioterapia Oncologica, Policlinico, Via del Pozzo 71, 41100, Modena, Italy; 5 U.O. Radioterapia, Istituti Ospitalieri, Largo Priori 1, 26100, Cremona, Italy; 6 U.O.C. Oncologia Radioterapica, IRCCS A.O.U. San Martino - Istituto Nazionale per la Ricerca sul Cancro, Largo Rosanna Benzi 10, 16132, Genova, Italy; 7 U.O. Radioterapia Oncologica “Giorgio Prodi”, Azienda Ospedaliera, Via Risorgimento 80, 42100, Reggio Emilia, Italy; 8 Divisione di Radioterapia, Istituto Europeo di Oncologia, Via Ripamonti 435, 20141, Milano, Italy; 9 U.O. Radioterapia Oncologica, Ospedale S.Chiara, Largo Medaglie d’Oro 9, 38100, Trento, Italy; 10 Divisione Universitaria di Radioterapia, Policlinico S.Orsola, Via Massarenti 9, 40138, Bologna, Italy; 11 S.C. Radioterapia “Ido Traldi”, Azienda Ospedaliera “Carlo Poma”, Viale Albertoni 1, 46100, Mantova, Italy

**Keywords:** Ductal carcinoma in situ (DCIS), Conservative surgery, Whole breast radiation therapy (RT), Risk factors for local recurrence

## Abstract

**Background:**

The incidence of ductal carcinoma in situ (DCIS) has increased markedly in recent decades. In the past, mastectomy was the primary treatment for patients with DCIS, but as with invasive cancer, breast-conserving surgery followed by radiation therapy (RT) has become the standard approach. We present the final results of a multi-institutional retrospective study of an Italian Radiation Oncology Group for the study of conservative treatment of DCIS, characterized by a very long period of accrual, from February 1985 to March 2000, and a median follow-up longer than 11 years.

**Methods:**

A collaborative multi-institutional study was conducted in Italy in 10 Radiation Oncology Departments. A consecutive series of 586 women with DCIS histologically confirmed, treated between February 1985 and March 2000, was retrospectively evaluated. Median age at diagnosis was 55 years (range: 29–84); 32 patients were 40 years old or younger. All women underwent conservative surgery followed by whole breast RT. Irradiation was delivered to the entire breast, for a median total dose of 50 Gy; the tumour bed was boosted in 295 cases (50%) at a median dose of 10 Gy.

**Results:**

After a median follow-up of 136 months (range: 16–292 months), 59/586 patients (10%) experienced a local recurrence: invasive in 37 cases, intraductal in 20 and not specified in two. Salvage mastectomy was the treatment of choice in 46 recurrent patients; conservative surgery in 10 and it was unknown in three patients. The incidence of local recurrence was significantly higher in women younger than 40 years (31.3%) (p= 0.0009). Five patients developed distant metastases. Furthermore 40 patients developed a contralateral breast cancer and 31 a second primary tumour in a different site. The 10-year actuarial overall survival (OS) was 95.5% and the 10-year actuarial disease-specific survival (DSS) was 99%.

**Conclusions:**

Our results are consistent with those reported in the literature. In particular it has been defined the importance of young age (40 years or less) as a relevant risk factor for local recurrence. This retrospective multi-institutional Italian study confirms the long term efficacy of breast conserving surgery with RT in women with DCIS.

## Background

Before screening programmes were introduced, breast ductal carcinoma in situ (DCIS) was a rather rare disease, accounting for 3-5% of breast tumours in the ‘70s-‘80s [1-3].

Its incidence is currently around 15-20% of breast carcinomas in Western countries [4,5]. Most cases (80-90%) are only diagnosed by mammography, whereas the clinical examination is negative; the mammographic pattern is often characterised by the presence of microcalcifications, at times associated with a mass [6].

Conservative surgery followed by radiation therapy (RT) to the residual breast is the standard treatment, as stated in the Consensus Conference [1] and the recent NIH State-of-the-Science Conference statement [7].

Four important randomised prospective trials [8-11] and several retrospective studies [12-16] have highlighted the importance of RT for the local control of DCIS, with an approximately 60% reduction in the relative risk of local recurrences [17].

In the subset of lesions with a very low risk of recurrence (small-sized, unicentric, low-grade tumours with adequate negative margins), some Authors [18-20] suggest omitting the complementary radiation treatment even though, to date, all subgroups of patients with DCIS have benefitted from RT. Even for women with negative margins and small low-grade tumours, the absolute reduction in the 10-year risk of ipsilateral breast events is 18% (2P= 0.002) in the analysis of the four randomized trials carried out by the Early Breast Cancer Trialists’ Collaborative Group [21].

At the beginning of the ‘90s, the scientific community’s interest in the study and evolution of the diagnostic and therapeutic approach to DCIS led to the creation of an *Italian Radiation Oncology Group for the study of conservative treatment of DCIS*, which published a first report in 1997 [22]. The present paper describes a second report of the Group on a larger number of patients with longer follow-up.

## Methods

We retrospectively evaluated a consecutive series of 610 patients with histological diagnosis of pure DCIS who were treated with conservative surgery and adjuvant RT from February 1985 through March 2000 in 10 Italian Hospitals.

Clinical, radiological and pathological data were collected in a central data-base at the Medical Oncology Department of Trento. The surgical approach consisted of quadrantectomy, or tumorectomy, or wide excision; axillary dissection was carried out in certain number of patients. Pathological assessment, including tumour size, margin status, histological subtype, nuclear grade and hormonal status, was obtained from the pathologic report of each patient. The surgical margin was considered positive if the tumour was reported at an inked margin, close if within < 2 mm and clear if ≥ 2 mm. The histological type was defined according to the traditional classification based on the architectural and morphological pattern of the tumour: comedo, cribriform, solid, papillary, micropapillary and mixed. Nuclear grade was determined according to the Consensus Conference on the Classification of Ductal Carcinoma In Situ [23]**.**

Postoperative RT was delivered by tangential fields encompassing the entire residual breast, using photons produced by a 60 Cobalt Unit or 4–6 MV photons of Linear Accelerators. The total dose ranged between 45 and 60 Gy (median: 50 Gy), in 1.8-2.5 Gy daily fractions (median: 2 Gy). Half of the patients also received a boost to the tumour bed, with a direct electron field with energy ranging between 6 and 12 MeV, or with Orthovoltage photons (energy: 150–300 KVp), or interstitial brachytherapy; the dose ranged between 8 and 20 Gy (median: 10 Gy).

Follow-up data on all patients was sought, including information on local and distant relapse. These data were obtained from medical record review in the Radiation Oncology or Medical Oncology Department where each patient had been followed. Local recurrences were subdivided into in-situ and invasive. Contralateral breast cancers were described as new primaries and distant metastases as any recurrence outside the breast and the regional lymph nodes. Additional data were searched in medical records of reference mammography Units and by asking demographic Departments for lost to follow-up patients.

In this case series an analysis was carried out on: incidence of local recurrences, distant metastases, second tumours (in the contralateral breast and other sites), overall survival (OS) and disease-specific survival (DSS).

The probability of local recurrence, OS and DSS were calculated by the Kaplan-Meier method [24], as from the date of surgery. Univariate analysis of the risk of local recurrence was performed, by means of Fisher’s exact test, to determine which of the following parameters were associated with local control: patient age (subdivided into 3 groups: ≤ 40, between 41–69 and ≥ 70 years), tumour size (< 10 mm, 10–30 mm, > 30 mm), nuclear grade (G1, G2, G3), surgical technique (quadrantectomy vs. tumorectomy vs. wide excision) and boost administration (yes vs. no). The cosmetic outcome, which was evaluated at the end of the treatment by the radiation oncologist at each of the participating Centres in the study, was rated as excellent, good, fair and poor, on the Harvard scale [25]. The cosmetic rate was not assessed by an independent reviewer.

## Results

**Figure 1:**
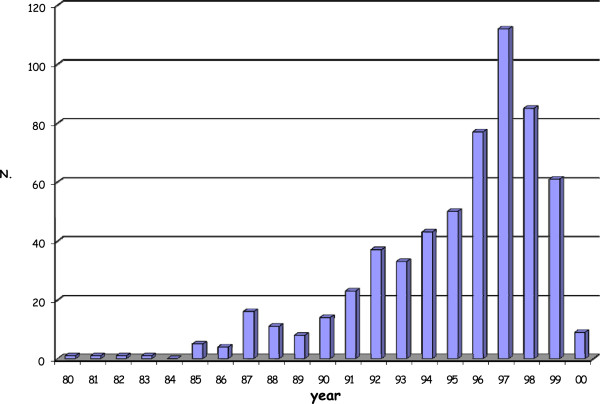
**Distribution of cases over the years (1985 – 2000).** Only approximately 20% of patients were treated in the first period (from 1985 to 1990) and the number of cases increased progressively during the following years.

From February 1985 to March 2000, 610 patients with histological diagnosis of pure DCIS were treated with conservative surgery followed by RT. The accrual on a yearly basis is shown in Figure 1.

Twenty-four patients were excluded from the analysis: 11 with a very short follow-up (< 12 months) and 13 owing to the presence of contralateral breast cancer before DCIS diagnosis. As a result, 586 patients were assessed.

The median follow-up was 136 months from the date of surgery, with a range of 16–292 months.

The main characteristics of the patients are shown in Table 1.

**Table 1:** Patient, tumour, and treatment characteristics

			
**Age**	Median	55 years	
	Range	29-84 years	
**Menopausal status**	Pre- or peri-menopause	181	31%
	Menopause	395	67%
	Unknown	10	2%
**Diagnosis modalities**	Mammography alone	387	66%
	Clinical signs and mammography	176	30%
	Clinical signs alone	23	4%
**Mammographic pattern**	Microcalcifications	393	67%
	Solitary mass	129	22%
	Microcalcifications + mass	41	7%
	Negative	23	4%
**Surgical management**	Quadrantectomy	434	74%
	Tumorectomy	88	15%
	Wide excision	64	11%
**Axillary dissection**	Yes*	240	41%
	No	346	59%
**Tumour pathological size**	≤ 10 mm	222	38%
	11-29 mm	203	34.5%
	≥ 30 mm	33	5.5%
	Unknown	128	22%
**Histological subtypes**	Comedo	164	28%
	Cribriform	164	28%
	Papillary	94	16%
	Solid	64	11%
	Other	100	17%
**Margin status**	Positive	4	1%
	Close	29	5%
	Negative	65	11%
	Unknown	488	83%
**ER/PR status**	Positive	253	43%
	Negative	64	11%
	Unknown	269	46%
**Radiotherapy technique**	60 Cobalt Unit	311	53%
	Linear Accelerator	275	47%
**Radiotherapy dose**	Median	50 Gy	
	Range	45 - 60 Gy	
**Boost**	No	291	49.5%
	Direct electron field	234	40%
	Orthovoltage photons	58	10%
	Interstitial brachytherapy	3	0.5%
**Boost dose**	Median	10 Gy	
	Range	8 - 20 Gy	

Some differences in the diagnostic, surgical and radiotherapeutic approach were observed comparing the first series of patients (from 1985 to 1990) with the complete series (from 1985 to 2000). In the first series the diagnosis was exclusively mammographic in 30% of cases; the axillary lymph node dissection was performed in 69% of women; a radiation boost to the tumour bed was delivered in 78% of patients. On the other hand in the complete series, 66% of cases were detected only by mammography; 41% of women underwent axillary dissection and the tumour bed was boosted in half of the patients.

A local recurrence was observed in 59 women (10%): the relapse was detected by mammography in 39 cases, by clinical signs in 10 cases, by both mammography and clinical signs in one patient, while in nine patients the detection modalities were unknown. In 40 cases it was a true recurrence, in the same quadrant; in 17 cases it was a recurrence in a different quadrant and in 2 cases the site was not specified. The histological diagnosis of the recurrence was carcinoma in situ in 20 cases (34%), invasive carcinoma in 37 cases (63%) and unknown in 2 cases (3%). All patients with a local failure had been previously treated with whole breast RT to a total dose of 50 Gy and 26 patients had also received a boost to the tumour bed. The administration of the boost did not significantly reduce the risk of local recurrences (p= 0.45), since the crude local recurrence rate was 10.8% and 8.9% among women who received boost and among those who did not, respectively.

The treatment of the recurrence was: salvage mastectomy with axillary lymph node dissection in 46 patients; further breast-conservative surgery in 10 patients, and not reported in 3 cases.

Two patients had axillary recurrences, after a local infiltrating recurrence. The therapeutic approach was: mastectomy with axillary lymph node dissection and chemotherapy in one case; irradiation of the axillary and supraclavicular region and chemotherapy in the other one. Both patients are alive, without disease progression.

**Figure 2:**
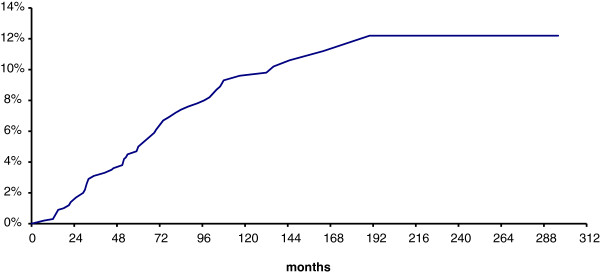
**Risk of local recurrence.** The risk of local recurrence was 5% at 5 years and 9.6% at 10 years.

The risk of local recurrence was 5% at 5 years and 9.6% at 10 years (Figure 2). The same figures were 4.8 % and 8.3 %, respectively, in patients given the radiation boost; 5.3 % and 11.5 %, respectively, in those not given the radiation boost. These differences were not statistically significant at the log-rank test. Only patient age resulted to be a statistically significant prognostic factor in the univariate analysis (p= 0.0009). Local recurrences occurred in 10/32 cases (31.3%) in women aged ≤ 40, in 46/517 cases (8.9%) in the age group between 41 and 69 years, and in 3/38 cases (7.9%) in women aged ≥ 70. The full results of univariate analysis are reported in Table 2. Since only one parameter achieved a statistical significance at the univariate analysis, multivariate analysis was not performed.

**Table 2:** Univariate analysis of risk factors related to local recurrence

**Parameter**	**p value**
Age	0.0009
Tumour size	0.06
Nuclear Grade	0.10
Surgical technique	0.51
Boost	0.45

Distant metastases were found in five patients (0.9%), who had previously developed a local infiltrating recurrence; 3 of these patients died because of the tumour and 2 are alive in progression with a follow-up of 203 and 206 months respectively.

In 40 patients (6.8%) a contralateral breast tumour was diagnosed (invasive in 30 cases; in situ in 9 cases and unknown in 1 case) and in 31 patients (5.3%) a second non-breast tumour (13 gynaecological, 6 gastroenteric, 3 hematologic, 2 pulmonary, 2 in the brain, 1 in the thyroid, 1 in the bladder and 3 cutaneous).

The actuarial overall 5-year and 10-year survival were 98.5% and 95.5%, respectively; the actuarial 5-year and 10-year DSS were 100% and 99%, respectively.

The cosmetic outcome, evaluated during the last follow-up visit by the radiation oncologist at each Centre, was excellent or good in 76% of the patients.

## Discussion

This multicentre retrospective study is characterised by a long period of accrual (median follow-up: 136 months,; range: 16–292 months), which offers the advantage of providing interesting information about the trend of the disease**.** A certain number of patients (12 patients) enrolled in the first years have died during this long period of observation; other patients (47 patients) didn’t return for the planned examination to the Centre of Radiotherapy where they had been treated, and were lost to follow-up. In addition only approximately 20% of patients were treated in the first period (from 1985 to 1990) and the number of cases increased progressively during the following years (Figure 1). Most of the cases belong to the two decades covered by the screening activity. Nevertheless, a large number of cases were also found in the younger age group, between 41 and 50. Increased debate and publicity about breast cancer screening after the introduction of screening programmes could have determined an improvement in women’s awareness, with a consequent increase of spontaneous mammography among age groups not invited for the screening, resulting in an increse of DCIS radiographycally detected [26].

In the entire case series, 66% of cases were identified by mammography, since the clinical pattern was negative, while in the series of patients treated between 1985 and 1990 the diagnosis was exclusively mammographic in only 30% of cases. These data show that, since the diffusion of screening, mammographic examinations have become widespread.

Quadrantectomy (74%) was the most frequent conservative surgery. In many cases, even among the oldest ones, x-ray examination of the surgical specimen or postoperative mammography was carried out to check completeness of the excision, as stated by the Consensus Conference [1].

A considerable number of cases (41%) were also subjected to axillary lymph node dissection. In the series of patients treated from 1985 to 1990, axillary lymphadenectomy was carried out in 69% of the cases. In the 1970s and 1980s most patients with DCIS underwent axillary lymph node dissection. During the 1990s the number of women undergoing axillary lymph node dissection for DCIS declined, in particular after the publication of the Consensus Conference [1]. Neither the indication to remove the axillary lymph nodes nor that to remove the sentinel lymph node is currently to be considered, except in the event of extensive, high-grade, palpable lesions, with a greater risk of occult invasion [1,27,28].

The total median dose of RT to the residual breast was 50 Gy with a 2 Gy/fr. conventional fractionation. Considering the entire case series, a boost to the tumour bed was also administered in 50% of the cases. In the analysis of the series treated in the 1985–1990 period, it was found that over 70% of the patients had received a boost. Our study showed no correlation between the prescription of the boost and the patients’ age and no significant difference was found in the distribution of local recurrences in relation to it (p= 0.45).

In the literature, the role of the radiation boost to the tumour bed has not been fully defined as yet. Some Authors have used a total dose of 10–20 Gy after standard whole breast RT [29-31]; or a total dose of 7.5 Gy in three fractions after standard or hypofractionated whole breast RT [32].

In the four randomised studies [8,11] that evaluated the role of postoperative RT after conservative surgery for DCIS , the boost was not envisaged.

The role of a higher dose on the initial site of the disease was evaluated in a retrospective analysis [33] on 373 patients ≤ 45 years old; it significantly reduced the risk of local recurrence (p < 0.0001). Besides the radiation boost, predictive factors for local recurrence included patients’ age (≤ 39 years) and the margin status. However, this study has some limits concerning its retrospective nature, the absence of a centralised review of histological samples and data on margin status, tumour size and grade. Randomised studies are underway and, while waiting for their results, it is advisable to administer the boost to young patients, in whom negative margins should always be obtained [1,7].

Considering that our study is retrospective, a comparative analysis of the results of the main retrospective studies [12-16] was performed. In our series, the incidence of local recurrences is 10%, and 63% of them are invasive. In the other series the incidence of local failures ranges between 8% and 20%, and around 50% (from 31% to 76%) are invasive. If one examines OS (98.5% at 5 years and 95.5% at 10 years) and DSS rates (100% at 5 years and 99% at 10 years), our data do not differ significantly from those reported in the literature [12-16].

The long period of accrual of this study has also caused some disadvantages.

The database sheets provided for the collection of the following pathological parameters: nuclear grade, margin status, estrogen and progesterone receptors, necrosis and multifocality, in addition to histological type and tumour size. The pathological records, especially those regarding the initial years of the study, did not always include all the above-mentioned parameters and, as it was not possible to review the specimens, they are unknown in a certain group of patients. We were able to carry out a detailed analysis of the risk for local recurrence only with regard to the following parameters: age of patients, tumour size and nuclear grade. Only age turned out to be a statistically significant prognostic factor (p= 0.0009).

Several prognostic factors correlated to the risk of local recurrence have been identified in the literature, such as clinical presentation, tumour size, histological type, nuclear grade and the presence of central necrosis, state and width of the margins and age of patients at diagnosis [12,34-39].

Young age (≤ 40 years) is considered to be one of the most important prognostic factors, in both the clinically palpable DCIS lesions [9,30,37-39] and the occult ones, diagnosed by mammography [14,40]. A number of clinical studies [9,14,41,42] report a local recurrence rate very similar to ours in the ≤ 40 age group.

In our study, most of the patients with local recurrence (78%) underwent mastectomy with axillary lymph-node dissection. The reasons for choosing breast-conserving surgery in 10 cases (16.9%) are unknown.

The therapeutic approach to local failure plays a major role, especially in the presence of an invasive recurrence. Mastectomy is the elective treatment for patients initially treated with conservative surgery and postoperative RT. In the case of small-sized and unifocal recurrences, local excision followed by RT is also suggested, if patients have not previously received RT [1,37]. The prognosis for in-situ recurrences is excellent, as less than 1% of patients develop further relapse, while it is much less favourable for invasive recurrences, which can develop metastases in 15-20% of the cases [31,43].

The use of accelerated partial breast irradiation (APBI) has been recently introduced not only in the treatment of invasive carcinoma at an early stage, but also of DCIS [44-46]. In 2005, a phase-III clinical trial comparing whole breast RT with APBI was conducted by means of one of the three following treatment techniques: multi-catheter interstitial brachytherapy, brachytherapy with MammoSite and 3D external conformal radiotherapy [44-47]. APBI experiences in the treatment of local recurrences are also reported in patients previously treated with conservative therapy (surgery followed by whole breast RT) for invasive breast carcinoma and DCIS [48,49]. In highly selected cases of local relapse, this therapeutic approach may find an important role in the future.

The incidence of distant metastases, after an invasive local recurrence, was 0.9% and the incidence of a second contralateral breast tumour was 6.8% in our series, in line with the results reported in the literature [17,50].

## Conclusions

This report represents one of the largest series of patients with DCIS treated in Italy over a long period of time, in highly specialized Centres regularly using a multidisciplinary approach to treat this disease.

Despite some differences in the therapeutic approach adopted by the 10 Centres participating in the study, with regard to the extension of the surgical excision, the use of axillary dissection and RT boost, the examined population is homogeneous and comparable to other published series.

The results, in terms of local and distant failures, second contralateral breast tumours as well as OS and DSS, are consistent with those reported in the literature. In particular, there emerges a significant increase in the risk of local recurrences in young women, reported by several Authors in both the clinical trials [8,9,11] and the retrospective studies [14,29,30,38,41].

Our study confirms the role played by postoperative RT in the conservative treatment of non invasive ductal carcinoma of the breast, as reported in numerous retrospective studies [12-16], in four prospective randomized clinical trials [8-11] and in three well-known metanalyses [17,21,50].

Studies of new irradiation modalities (PBI, hypofractionation) in DCIS patients are currently ongoing and deserve further attention.

## Competing interests

The authors declare that they have no competing interests.

## Authors’ contributions

OC performed the statistical analysis for the study. CV participated in the design and coordination of the study. All authors collected and analyzed patient data, and drafted the manuscript. All authors read and approved the final manuscript.
